# Amperometric Biosensor Based on Glutamate Oxidase to Determine Ast Activity

**DOI:** 10.3390/s24247891

**Published:** 2024-12-10

**Authors:** Daryna Mruga, Kseniia Berketa, Andrii Sverstiuk, Vasyl Martsenyuk, Aleksandra Klos-Witkowska, Yurii Palianytsia, Sergei Dzyadevych, Oleksandr Soldatkin

**Affiliations:** 1 Institute of Molecular Biology and Genetics, National Academy of Sciences of Ukraine, 03680 Kyiv, Ukraine; darynamruga@gmail.com (D.M.); dzyad@yahoo.com (S.D.); alex_sold@yahoo.com (O.S.); 2 Institute of Biology and Medicine, Taras Shevchenko National University of Kyiv, 01003 Kyiv, Ukraine; 3 Department of Medical Informatics, I. Horbachevsky Ternopil National Medical University, 46002 Ternopil, Ukraine; 4 Department of Computer Sciences, Ternopil National Ivan Puluj Technical University, 46001 Ternopil, Ukraine; 5 Department of Computer Science and Automatics, University of Bielsko-Biala, 43309 Bielsko-Biala, Poland; vmartsenyuk@ath.bielsko.pl (V.M.); awitkowska@ath.bielsko.pl (A.K.-W.); 6 Radio Engineering Systems Department, Ternopil National Ivan Puluj Technical University, 46001 Ternopil, Ukraine; palanizayb@tntu.edu.ua; 7 Faculty of Biomedical Engineering, Igor Sikorsky Kyiv Polytechnic Institute, 03056 Kyiv, Ukraine

**Keywords:** amperometry, aspartate aminotransferase, biosensor, glutamate oxidase, immobilized enzyme, measurement of aspartate aminotransferase activity

## Abstract

This work presents the development of an amperometric biosensor for detecting aspartate aminotransferase (AST) activity in biological fluids using a platinum disk electrode as the working transducer. Optimal concentrations of substrates (aspartate, α-ketoglutarate) and the coenzyme (pyridoxal phosphate) were determined to ensure efficient biosensor operation. A semi-permeable poly-m-phenylenediamine membrane was applied to enhance selectivity against electroactive interferents. The biosensor demonstrated good stability (storage, continuous operation, and production reproducibility) and analytical performance (sensitivity 8.56 nA/min for 50 U/L AST, LOD 1 U/L, linear range 1–110 U/L). Testing with real samples showed a high correlation (R = 0.989) with spectrophotometric analysis, supporting its potential for further applications.

## 1. Introduction

According to the WHO [1], the most common cause of human death worldwide is cardiovascular diseases, particularly ischemic heart disease. Therefore, monitoring the health of the cardiovascular system is highly relevant today. One of the most common biomarkers of myocardial damage is the organ-specific cytoplasmic enzyme aspartate aminotransferase (AST), which is typically present in low concentrations in the blood of healthy individuals (up to 32 U/L in men and up to 26 U/L in women [2]). However, when myocardial cells are damaged, intracellular contents are released into the intercellular space or directly into the blood, leading to a significant increase in AST activity (up to 2000 times) [3,4,5], which correlates with the severity of the damage. Elevated AST levels are also commonly associated with liver damage [6,7,8].

Over the years, various methods for measuring AST in blood serum have been developed. The spectrophotometric method remains the most widely used approach in commercial diagnostic kits due to its simplicity, well-established protocols, and widespread availability [9,10,11,12]. Despite its advantages, spectrophotometry has several limitations, including the need for bulky, expensive equipment and its lack of adaptability to field conditions where rapid, real-time measurements are required. Moreover, spectrophotometry is not suitable for portable or point-of-care diagnostics, which is especially important for patients with chronic cardiovascular diseases or in emergency situations.

Recently, several alternative methods for AST detection have emerged, driven by the need for portable and more efficient diagnostic systems. Among these, immunoassays [13,14] have shown promise due to their high specificity, but they still face challenges in terms of cost and the need for specialized equipment. Optical biosensors [15,16,17,18,19], mostly based on the detection of light absorption, have also been explored as potential alternatives, offering the advantage of real-time detection and easy sample preparation. However, these methods often suffer from a low detection range, and there are strict requirements for conducting analysis under certain conditions.

In contrast, electrochemical biosensors [20,21,22,23] have gained significant attention in recent years due to their simplicity, low cost, and ease of integration into portable devices. Electrochemical biosensors can be designed to detect AST through amperometric or potentiometric methods, providing high sensitivity and fast response times. These sensors often employ enzymes such as glutamate oxidase, oxaloacetate decarboxylase, and horseradish peroxidase. Despite their promising potential, challenges remain in achieving the required sensitivity, selectivity, and stability for reliable clinical applications.

Currently, laboratory prototypes of electrochemical biosensors for AST detection have demonstrated performance comparable to or even better than traditional methods such as spectrophotometry. However, they have not yet reached the level of reliability and commercial acceptance required for widespread clinical use.

Therefore, the development of a reliable, highly sensitive, and easy-to-use biosensor for AST-level determination remains an important goal in advancing point-of-care diagnostics for cardiovascular and liver diseases. The creation of a biosensor system for the simultaneous measurement of AST and ALT levels is an even more relevant task due to its higher diagnostic value and capability for the more thorough monitoring of patient health. Therefore, the purpose of this study was to develop an amperometric biosensor based on glutamate oxidase for measuring AST content in biological fluids, which can operate both independently and in combination with biosensors for ALT determination.

## 2. Materials and Methods

### 2.1. Materials

Materials for the bioselective membrane: recombinant glutamate oxidase (GlOx) from Streptomyces sp. with an activity of 7 U/mg (Yamasa Corporation, Choshi, Japan); bovine serum albumin (BSA), glutaraldehyde (GA), and glycerol were from Sigma-Aldrich, Inc. (St. Louis, MO, USA).

Analyte and substrates: aspartate aminotransferase (AST) from the porcine heart with an activity of 188 U/mg protein, pyridoxal phosphate (PLP), L-aspartate, α-ketoglutarate (α-KG), sodium L-glutamate, HEPES were from Sigma-Aldrich, Inc. H_2_O_2_ was from Logic Lab Group, Kyiv, Ukraine.

Materials for additional membrane: meta-phenylenediamine was from Sigma-Aldrich, Inc.

Interferents: alanine aminotransferase (ALT) from the porcine heart with an activity of 75 U/mg protein, ascorbic acid, uric acid, citric acid, benzoic acid, other amino acids, acetaminophen, glucose were from Sigma-Aldrich, Inc. KCl, CaCl_2_, and NaN_3_ were from Logic Lab Group (Kyiv, Ukraine).

Other materials: ethanol and powder of aluminum oxide were from Logic Lab Group.

### 2.2. Design of the Amperometric Transducer and Scheme of the Experimental Setup

In this work, a platinum disk electrode with a working area of 0.126 mm^2^ was used as an amperometric transducer. All electrodes used in this work were manufactured at the Institute of Molecular Biology and Genetics of the National Academy of Sciences of Ukraine. The principle of their production and detailed design was described in a previous work [24].

A three-electrode scheme of amperometric analysis was used. The working electrodes, a counter platinum electrode, and an Ag/AgCl reference electrode were connected to a PalmSens potentiostat (Palm Instruments BV, CL Houten, The Netherlands). All of the mentioned electrodes were connected to the potentiostat through an 8-channel PalmSens multiplexer, which allowed signals to be received from several working electrodes at the same time.

### 2.3. The Method of Applying an Additional Membrane Based on PPD

A semi-permeable additional membrane made of poly-(meta-phenylenediamine) was formed on the surface of the electrochemical transducer. This membrane formed a mesh-like structure that served as a physical filter for substances that could reach the charged electrode space. Thus, electroactive interfering substances could not reach the working surface of the transducer and did not cause a non-selective response in the system. This membrane was formed according to the following method: the working electrode was immersed in a 4 mM solution of m-phenylenediamine in a 5 mM phosphate buffer, and 20 cycles of cyclic voltammetry from 0 to 0.9 V with a step of 0.05 V/s were performed. Stable indicators of the voltammogram across different cycles confirmed the quality of the electropolymerization. Subsequently, the sensor was washed in a working buffer for 10 min.

### 2.4. The Method of Applying a Bioselective Membrane

The bioselective membrane on the surface of the electrochemical transducer was formed by the method of covalent binding on the enzyme in a mesh-like three-dimensional structure. The mesh structure was formed by crosslinks between protein molecules. Glutaraldehyde (GA), which is able to form covalent bonds with two amino groups, was used as a crosslinking agent.

Enzyme gel (8% GlOx, 4% BSA, 10% glycerol in 100 mM PBS, pH 6.5) and crosslinker (0.5% aqueous solution of GA) were mixed in a 1:2 ratio. Approximately 50 nL of this mixture was deposited on the sensitive area of the working electrode and air-dried at RT for 35 min. Then, the biosensor was washed from non-bound molecules in a working buffer solution (25 mM HEPES, pH 7.4) for 10 min. The final membrane consisted of 53 g/L of GlOx, 13 g/L of BSA, 33 g/L of glycerol, and 3.3 g/L of GA (see optimization in Supplementary Figures S1 and S2).

### 2.5. Method of Measuring AST Content in Model Solutions and Real Samples

In this study, AST content was determined by measuring its activity, specifically through the rate of formation of reaction products, which is proportional to AST activity under conditions of full saturation. The rate of product formation was monitored by measuring changes in the current within the system.

Biosensor measurements were conducted at room temperature in a 2 mL open measuring cell with uniform stirring and a constant potential of +0.6 V against the Ag/AgCl reference electrode, which aligns with the optimal oxidation potential of H₂O₂ on a platinum anode. A 25 mM HEPES buffer (pH 7.4) was used as the working solution, and substrate concentrations were adjusted by adding aliquots of concentrated solutions.

Each experiment was repeated 3–4 times, and the data presented represent the average values. Calculations were performed using OriginLab OriginPro 8.5 software.

For real samples, a 10-fold serum dilution was applied. The AST content was then calculated using the calibration curve method: a linear equation was generated to describe the biosensor’s response to AST activity, and AST activity in the sample was subsequently calculated using this equation, the recorded biosensor response, and the dilution factor.

### 2.6. Method of Spectrophotometric AST Detection

Reference analysis was performed on a SPECORD 50 PLUS spectrophotometer (Analytik Jena, Jena, Germany). The principle of detection was as follows: AST catalyzes the reaction of transamination resulting in the formation of glutamate; glutamate is oxidized by glutamate oxidase with the formation of hydrogen peroxide; hydrogen peroxide participates in the peroxidase-catalyzed reaction between N-Ethyl-N-(2-hydroxy-3-sulfopropyl)-m-toluidine and 4-aminoantipyrine that results in the production of a colored substance (absorbance maximum at 552 nm). The speed of dye formation correlates with AST content. The procedure for real samples was as follows: 100 µL of the sample was mixed with 900 µL of 25 mM HEPES solution with reagents (final content in the cuvette was 8 mM aspartate, 2 mM α-ketoglutarate, 0.25 U/mL GlOx, 2420 U/mL peroxidase, 0.66 mM EHSPT, 0.48 mM 4-aminoantipyrine, and different concentrations of additional AST to imitate pathologic levels). The changes in the optical density of the sample were measured for 15–16 min after mixing. The response was received as ∆ optical density per minute.

## 3. Results and Discussion

### 3.1. The Principle of the Biosensor Operation

In order to register biochemical processes using amperometry, the formation of electroactive substances during these processes is necessary. Given that the enzymatic reaction catalyzed by AST does not involve electroactive substances, it is difficult to detect this reaction using an electrochemical transducer. Therefore, to detect AST activity in the solution, we chose to monitor the oxidation of one of AST’s reaction products, glutamate, which leads to the formation of electroactive hydrogen peroxide. The enzymatic reactions underlying the function of this amperometric biosensor for AST detection are shown in Reactions (1)–(4).

AST: aspartate + PLP ↔ oxaloacetate + PLP_NH4_ (1)

AST: α-ketoglutarate + PLP_NH4_ ↔ glutamate + PLP (2)

GlOx: glutamate → α-ketoglutarate + NH_3_ + H_2_O_2_ (3)

600 mV: H_2_O_2_ → e^−^ + H^+^ + O_2_ (4)

Hence, the sustained activity of AST leads to an increase in glutamate concentration, which is subsequently converted into hydrogen peroxide with the assistance of immobilized GlOx (see kinetics in Supplementary Figure S3). Under the applied potential, hydrogen peroxide undergoes oxidation on the surface of the platinum electrode, resulting in a current increase within the system, detected by the amperometric sensor. The content and units of activity of AST in the measuring cell correlates with the rate of current change in the system. For ease of calculation, a response time of 1 min was selected.

**Figure 1:**
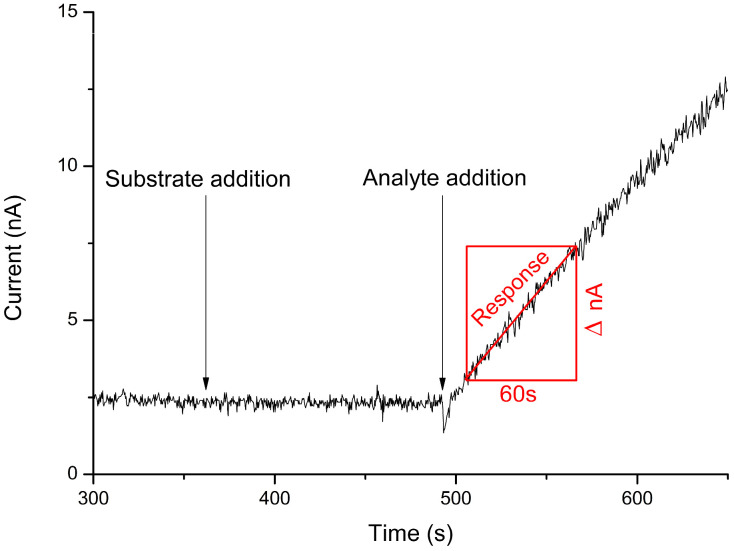
Typical response of the biosensor to the introduction of AST into the measuring cell. Measurements were performed in a 25 mM HEPES buffer at pH 7.4, at a constant potential of +0.6 V relative to the Ag/AgCl reference electrode at room temperature.

Thus, a typical experiment proceeded as follows (Figure 1): the sensor’s baseline was established, followed by the addition of an aliquot containing AST substrates (8 mM aspartate and 2 mM α-ketoglutarate) into the measuring cell and, subsequently, an aliquot of AST was pipetted into the solution. Upon the introduction of the enzyme, there was a notable increase in current within the system. The acquired data were then linearized to enhance accuracy, and the response was quantified as ∆I/minute.

### 3.2. Selection of Optimal Concentrations and Ratios of AST Substrates

**Figure 2:**
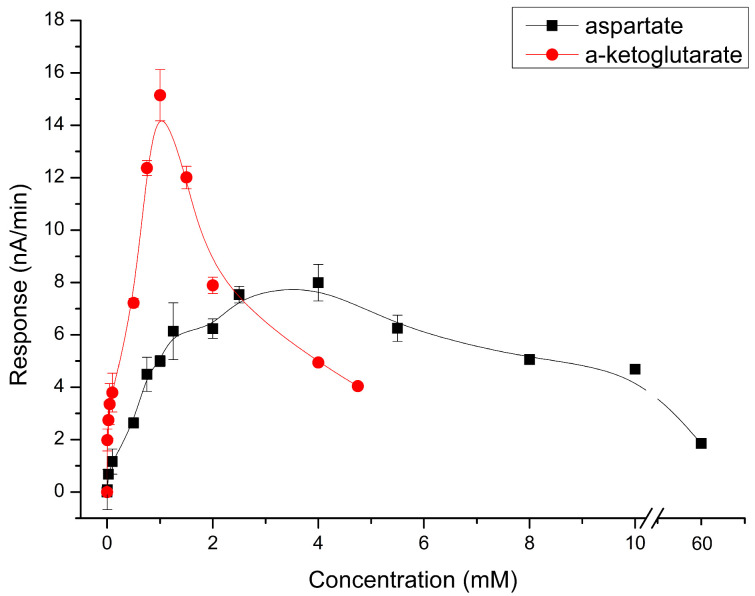
Dependence of the response value of the biosensor on 100 U/L of AST on the concentration of aspartate and α-ketoglutarate. Measurements were performed in a 25 mM HEPES buffer, pH 7.4, at a constant potential of +0.6 V relative to the Ag/AgCl reference electrode at room temperature.

Considering that the proposed biosensor determines the AST activity based on the dynamics of changes in the reaction product concentration, it is crucial to ensure the enzyme operates at its maximum possible speed. For the most efficient reaction, a constant supply of substrates and the release of products from the enzyme must be ensured. AST utilizes two substrates with varying degrees of affinity for the same active site, leading to competitive inhibition in their suboptimal ratio. In the study, the optimal ratio of substrates was determined (Figure 2). Initially, the concentration of aspartate was assessed to facilitate the subsequent determination of the optimal substrate concentration ratio by selecting the optimal concentration of α-ketoglutarate. This ratio was four to one (in this case, 4 mM aspartate to 1 mM α-ketoglutarate). This difference can be explained by a stronger complex of AST-α-KG and the fact that the concentration of α-KG is partially restored due to the work of glutamate oxidase.

**Figure 3:**
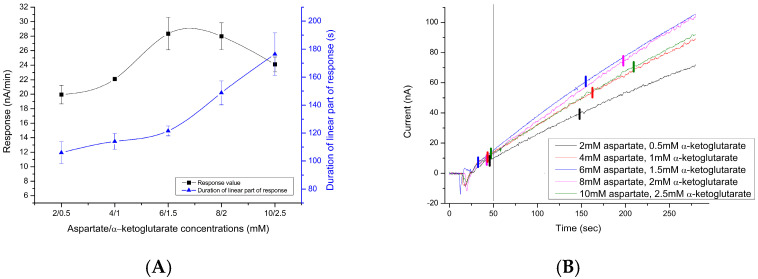
Dependence of the response value and duration of the linear part of the response of the biosensor to 100 U/L of AST on the concentrations of the substrates (**A**). Four responses of the biosensor to the introduction of 100 U/L of AST into the measuring cell in the presence of different concentrations of substrates (aspartate and α-ketoglutarate in a ratio of 4:1) (**B**). Vertical lines at 50 and 110 s mark the limits of response removal; small vertical lines further mark the beginning and end of response linearity. Measurements were performed in a 25 mM HEPES buffer, pH 7.4, at a constant potential of +0.6V relative to the Ag/AgCl reference electrode at room temperature.

However, for the maximum speed of the enzyme, not only the probability of receiving substrates in the correct order (ratio) but also the speed of their arrival plays a role. Substrates are delivered to the active center by passive diffusion; accordingly, to speed up this process, it is necessary to increase their concentration. However, excessive substrate content can lead to substrate inhibition. Therefore, the optimal concentrations of substrates were determined while maintaining their ratio of 4:1 (Figure 3A). As can be seen from the experiment, such optimal concentrations were 6–8 mM aspartate and, accordingly, 1.5–2 mM α-ketoglutarate.

Since there is a possibility that when using a biosensor for the analysis of real samples, the method of “standard additions” will have to be used instead of the method using a calibration curve, in addition to the magnitude of the response, the linearity of this response plays an important role. In general, there is a tendency to lengthen the duration of the linearity of the response with an increased concentration of AST substrates (Figure 3B). For this work, it was decided to use concentrations of 8 mM aspartate and 2 mM α-ketoglutarate since such concentrations allow the best ratio to be obtained between the magnitude of the response and the duration of the linear range.

### 3.3. Selection of the Optimal Coenzyme Concentration

**Figure 4:**
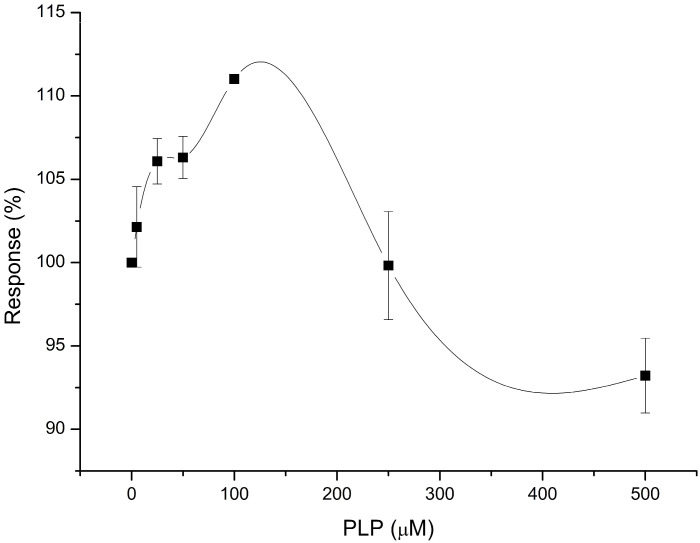
Dependence of the response value of the biosensor on 100 U/L of AST on different concentrations of PLP in the measuring cell. Concentrations of substrates in the solution are 8 mM aspartate and 2 mM α-ketoglutarate. Measurements were performed in a 25 mM HEPES buffer, pH 7.4, at a constant potential of +0.6 V relative to the Ag/AgCl reference electrode at room temperature.

According to the literature, AST works in the presence of the coenzyme pyridoxal phosphate (PLP). PLP acts as an intermediate amino group acceptor between successive interactions of the AST active center with substrates. Considering this, it was decided to examine the influence of different concentrations of PLP in the working buffer on the value of the responses of the developed biosensor to the AST introduced (Figure 4). The expected result was an increase in the efficiency of AST and, accordingly, an increase in the response magnitude in the presence of PLP. Experimental data showed that the presence of PLP in a concentration greater than 100 μM caused a decrease in the magnitude of the response to AST. This can be explained by the fact that a high concentration of PLP accelerates the exchange rate of the coenzyme molecules between the enzyme and the solution (reaction (5)).

PLP_NH4_-AST + PLP ↔ PLP_NH4_ + AST-PLP (5)

Thus, PLP with an amino group detaches from the active center of AST, thereby blocking the course of reaction (2), during which the intermediate analyte of this biosensor—glutamate—is formed. At a lower concentration (100 µM), PLP causes an increase in the value of the biosensor’s response to AST, but its effect is smaller than expected (less than 12%).

### 3.4. Study of Biosensor’s Selectivity

Since this biosensor is intended for further use to measure the level of AST in the blood serum, it is necessary to take into account the influence of interferents present in biological fluids on its operation. For this, the selectivity of the developed biosensor towards substances of different natures was checked (Table 1).

It is known that the presence of electroactive substances usually causes the response of the amperometric sensor in the form of a current jump in the system followed by a gradual return of the signal to the baseline due to the electrochemical oxidation on the working electrode and, accordingly, a decrease in the concentration of these substances on the working buffer. This can cause a decrease in the biosensor’s response value due to oppositely directed processes: 1—a drop in current strength due to the oxidation of interferents; 2—an increase in current strength due to an increase in the glutamate concentration caused by AST activity. Therefore, it was decided to reduce the influence of the electroactive substances by applying an additional poly(m-phenylenediamine) (PPD) membrane [25] between the bioselective membrane and the electrode surface. This resulted in the physical blocking of large molecules’ access to the surface of the transducer and, consequently, an improvement in its selectivity (see Supplementary Figure S4).

It was also determined that non-electroactive substances, potentially present in the target biological fluids at concentrations up to 1 mM, did not elicit a response from the GlOx-based biosensor and did not affect the response magnitude to glutamate or AST.

In addition, the selectivity of the biomembrane to the intermediate analyte (glutamate) among other amino acids was tested. It was determined that some free amino acids (aspartic acid, asparagine, histidine, glutamine) caused the responses from the biosensor were two orders of magnitude lower than the response to glutamate. Sensitivity to other amino acids was not observed. However, since the biosensor’s response depends not on the immediate concentration of glutamate but on its rate of change over time, the influence of other amino acids introduced with the sample could be disregarded.

In addition, it is known that an increase in the AST blood level is often accompanied by an increase in the level of ALT. Since these enzymes are from the same family, have similar reaction patterns, and even share the same second reaction step, there is a possibility that the presence of ALT could affect the response of the biosensor. Therefore, this assumption was tested, and it was established that the presence of ALT (in the same concentration as AST) did not affect the biosensor’s performance.

### 3.5. Study of the Stability of the AST-Sensitive Biosensor

**Figure 5:**
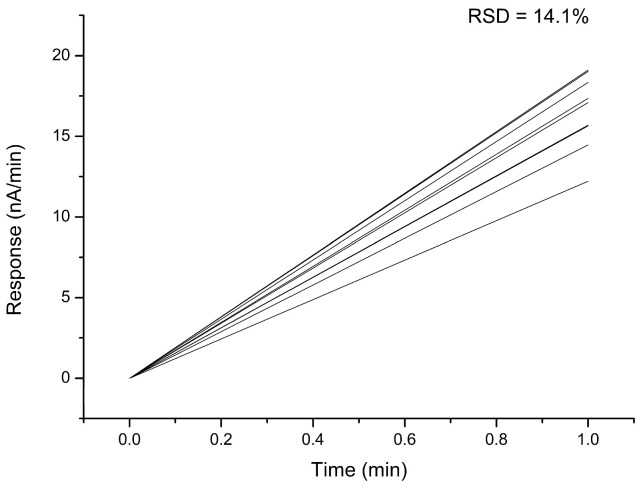
Reproducibility of the biosensor’s responses to the addition of 100 U/L AST to the measuring cell. Substrate concentrations of 4 mM aspartate, 50 µM α-ketoglutarate, and 50 µM PLP. Measurements were performed in a 25 mM HEPES buffer, pH 7.4, at a constant potential of +0.6 V relative to the Ag/AgCl reference electrode at room temperature.

For an accurate measurement of the analyte content in the analyzed sample using a biosensor, the high reproducibility of its responses is essential. Therefore, it was decided to evaluate this parameter for the biosensor developed in this work. To achieve this, 10 measurements of the same AST concentration were conducted during a single working day. It was determined that the root mean square deviation of the biosensor responses to AST was 14.1% (Figure 5).

**Figure 6:**
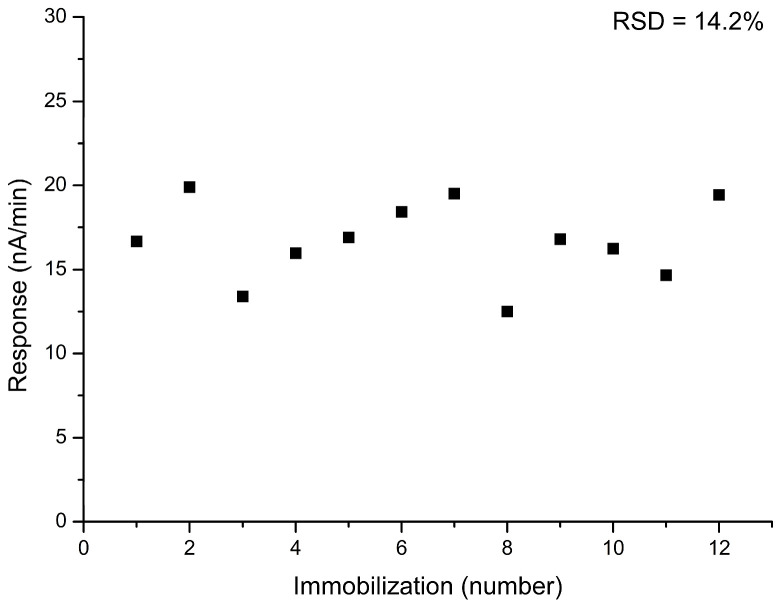
Reproducibility of the preparation procedure of a biosensor based on GlOx. The AST concentration was 100 U/L. Substrate concentrations were 8 mM aspartate and 2 mM α-ketoglutarate. Measurements were performed in a 25 mM HEPES buffer, pH 7.4, at a constant potential of +0.6 V relative to the Ag/AgCl reference electrode at room temperature.

Another important parameter affecting the possibility of further metrological attestation and practical implementation is the reproducibility of the enzyme biosensor preparation procedure. This parameter assesses the variability in sensitivity to the analyte within the batch of biosensors. To evaluate this, 12 biosensors were created using the same manufacturing process. Their sensitivity to AST was tested, and the error was calculated. The average statistical deviation of biosensors’ responses during production was 14.2% (Figure 6). This variability was likely due to the manual application of the membrane onto the transducer surface, which can cause differences in membrane thickness and uniformity. It is expected that transitioning to an automated membrane application process will enhance precision and consistency, thereby reducing this error and improving the overall performance and reproducibility of the biosensor.

The stability of the biosensor when stored in a freezer (−18 °C) was also evaluated. After two months of storage, the activity of the developed biosensor based on GlOx remained at 70% of its initial activity. Providing additional calibration, the proposed biosensor could be successfully used to further determine AST activity in solution.

### 3.6. Analytical Characteristics of the Biosensor for AST Determination

**Figure 7:**
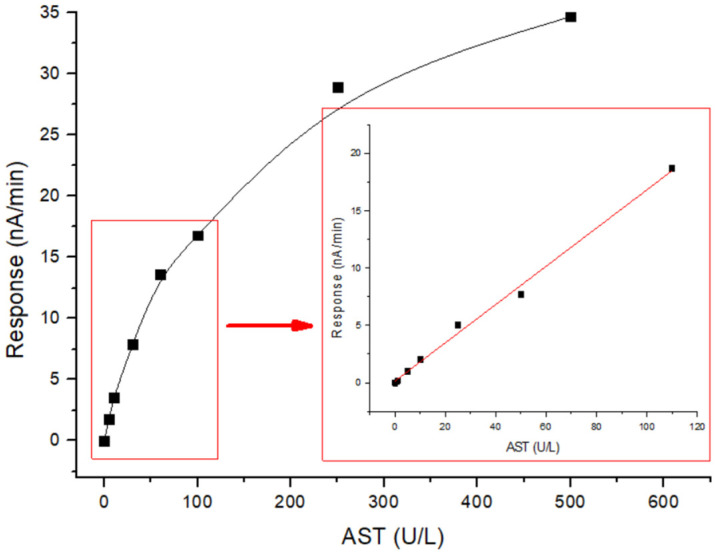
Calibration curve of the biosensor for the determination of AST and its linear section. Substrate concentrations were 8 mM aspartate and 2 mM α-ketoglutarate. Measurements were performed in a 25 mM HEPES buffer at pH 7.4, at a constant potential of +0.6 V relative to the Ag/AgCl reference electrode at room temperature.

The developed biosensor was characterized by a wide dynamic range of the determination of AST content (1–500 U/L) and a linear range (1–110 U/L), which is sufficient for determining normal and pathological levels of this enzyme in blood serum. Figure 7 shows the overall calibration curve of the biosensor for AST determination and highlights the detailed linear part of this curve (∆I = 0.16703 ∗ CAST + 0.14202). From this, it can be seen that the sensitivity of the proposed biosensor was 8.56 nA/min per 50 U/L of AST. The minimum detection limit was 1 U/L, and the linear segment of the response lasted 160 s. The duration of the entire analysis, including the preparation, measurement, and processing of the results, was approximately 15 min. In conclusion, an amperometric biosensor based on GlOx was developed to determine the AST content, and its main analytical characteristics were evaluated. The obtained data indicate the prospects of the further application of this biosensor for measuring AST activity in real blood serum samples.

### 3.7. Testing of the Developed AST-Sensitive Biosensor with Real Serum Samples

**Figure 8:**
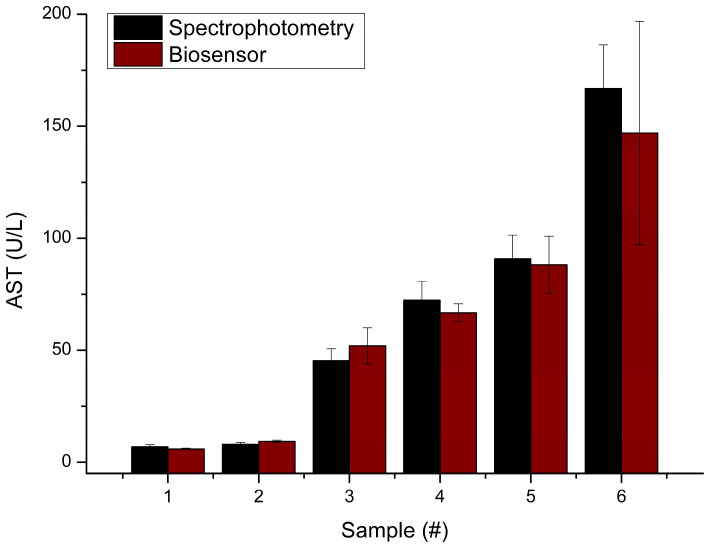
Comparison of spectrophotometric and biosensor analyses of serum samples. The difference between groups was non-significant (*p*-value > 0.05).

For the further exploitation of the developed biosensor for the detection of the AST activity level in biological fluids, it was necessary to verify its accuracy by comparing the results of the biosensor and reference analysis of real samples. To achieve this, AST content was analyzed in six blood serum samples, with analyte levels within the normal range (6–40 U/L AST) and significantly elevated levels in pathological cases (up to 180 U/L AST). The measurement procedures for both methods are described in the Section 2. The obtained results showed a correlation coefficient of 0.989 between the biosensor and reference method results (Figure 8), which is a strong indicator of the accuracy and reliability of the biosensor method.

## 4. Conclusions

This study focuses on the development of an amperometric biosensor based on glutamate oxidase for the selective determination of AST content in aqueous solutions, particularly the blood serum. During development, the optimal concentrations of AST substrates (aspartate and α-ketoglutarate) were determined, and the impact of the AST coenzyme (PLP) on biosensor performance was evaluated. The selectivity of the biosensor was assessed for structurally and functionally similar enzymes (e.g., ALT), as well as for amino acids and neutral and electroactive substances that may be present in biological fluids, demonstrating minimal interference from these compounds.

The biosensor’s analytical characteristics were thoroughly investigated, including its dynamic and linear ranges, minimum detection limit, sensitivity, duration of linear response, analysis time, reproducibility of responses, reproducibility of preparation, and storage stability. The developed biosensor exhibited high sensitivity and a sufficient linear range for measuring both normal and pathological AST levels in blood serum. Validation using the spectrophotometric reference analysis of serum samples confirmed the biosensor’s accuracy, with a correlation coefficient of 0.989 between the two methods.

Future research will focus on optimizing the biosensor for use with undiluted blood serum samples and developing a multi-analyte biosensor system for the simultaneous detection of AST and ALT, both of which are key biomarkers of heart and liver damage.

## Figures and Tables

**Table 1:** Study of the selectivity of the developed biosensor towards non-target substances.

Substance, 1 mM	Response nA	Substance, 1 mM	Response nA	Substance, 1 mM	Response nA
Electroactive					
Ascorbic acid	2.00	Cysteine	0	Uric acid	0
Dopamine	1.20	Citric acid	0		
Non-electroactive					
EDTA	0	CaCl_2_	0	PLP	0
KCl	0	glucose	0	Acetaminophen	0
NaN_3_	0	NaCl	0	α-Ketoglutarate	0
Benzoic acid	0				
Amino acids					
Glutamate	175.00	Aspartate	0.30	Histidine	0.20
Asparagine	0.90	Leucine	0.05	Tyrosine	0.15
Glutamine	0.35	Valine	0	Methionine	0.10
Arginine	0	Isoleucine	0	Lysine	0
Threonine	0	Cystine	0	Proline	0
Alanine	0	Serine	0		

## Data Availability

Data will be provided upon request.

## Supplementary Materials

The following supporting information can be downloaded at https://www.mdpi.com/1424-8220/24/24/7891/s1. Figure S1: Dependence of response on the ratio between volumes of enzyme solutions and GA. Measurements were carried out in 25 mM HEPES buffer, pH 7.4, at a constant potential of +0.6 V vs. Ag/AgCl reference electrode; Figure S2: Dependence of the biosensor response on the GA concentration in the immobilization solution. The glutamate concentration 1000 μM. Measurements were carried out in 25 mm HEPES buffer, pH 7.4, at a constant potential of +0.6 V vs. Ag/AgCl reference electrode; Figure S3: Michaelis-Menten equation direct fitting of the data obtained from bioselective element (A) and the Lineweaver-Burk plot of the mentioned data (B); Figure S4: Responses of the biosensor without (A) and with (B) PPD membrane to the addition of interferents. Measurements were carried out in 25 mm HEPES buffer, pH 7.4, at a constant potential of +0.6 V vs. Ag/AgCl reference electrode.
