# Supplementary material for: Amperometric Biosensor Based on Glutamate Oxidase to Determine Ast Activity
Source: Sensors (Basel). 2024 Dec 10;24(24):7891. doi: 10.3390/s24247891 (PMC11840281; doi:10.3390/s24247891)
Supplement: Supplementary file 1 [file sensors-24-07891-s001.zip › sensors-3337082-supplementary.pdf]

AMPEROMETRIC BIOSENSOR BASED ON GLUTAMATE OXIDASE FOR DETERMINATION OF AST ACTIVITY

SUPPLEMENTARY

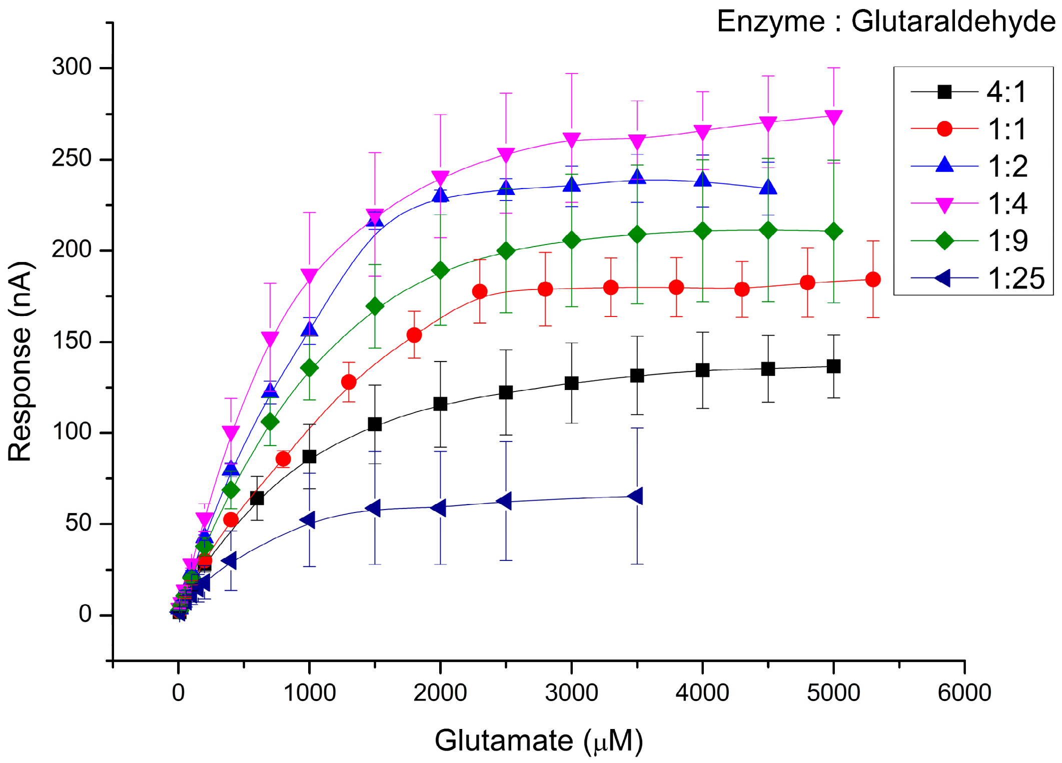

Figure S1. Dependence of response on the ratio between volumes of enzyme solutions and GA. Measurements were carried out in 25 mM HEPES buffer, pH 7.4, at a constant potential of +0.6 V vs Ag/AgCl reference electrode.

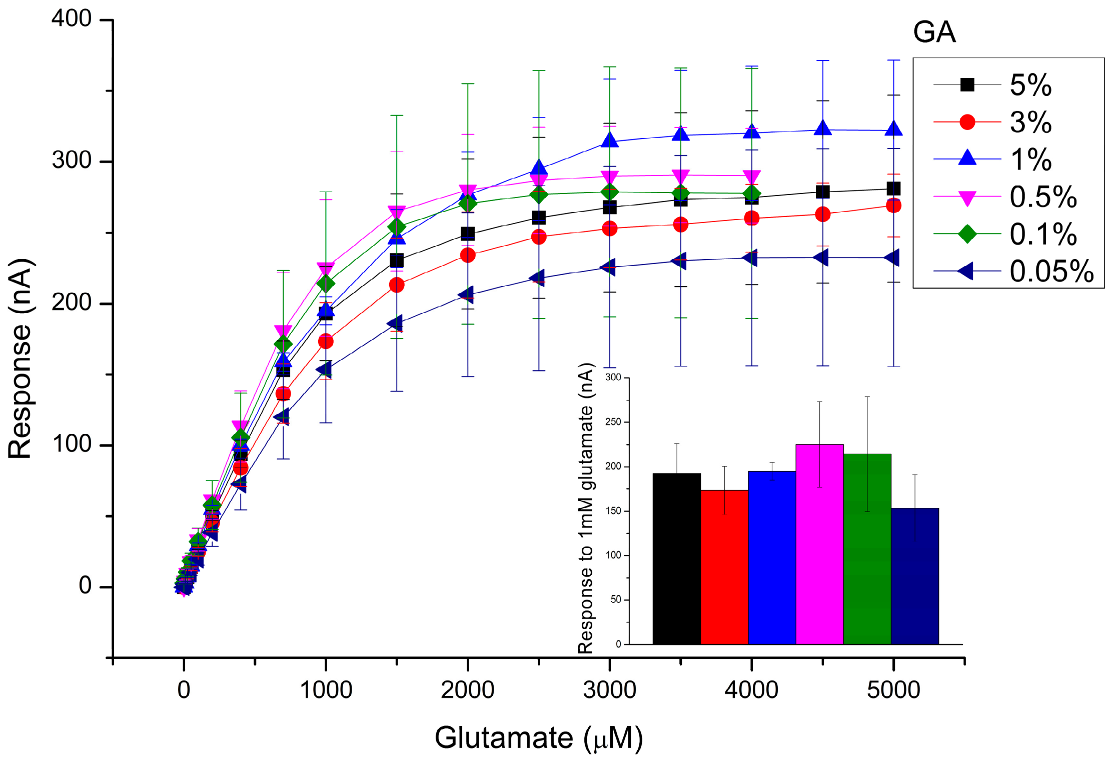

Figure S2. Dependence of the biosensor response on the GA concentration in the immobilization solution. The glutamate concentration - 1000 μM. Measurements were carried out in 25 mm HEPES buffer, pH 7.4, at a constant potential of +0.6 V vs Ag/AgCl reference electrode.

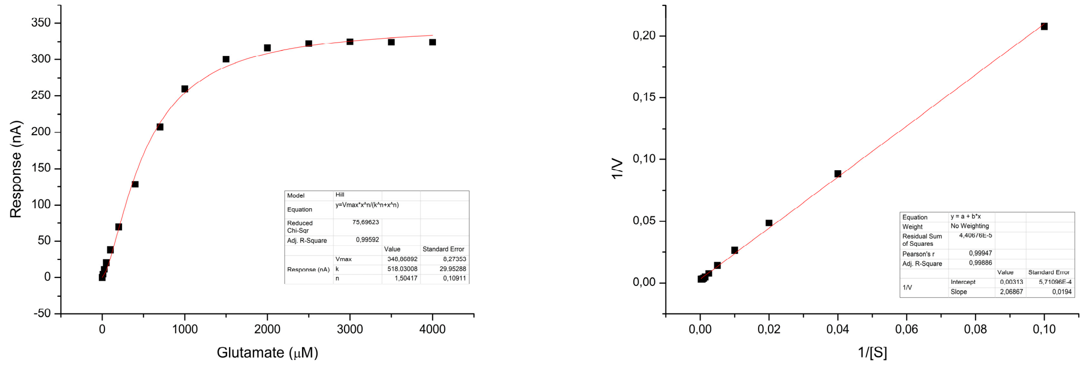

Figure S3. Michaelis-Menten equation direct fitting of the data obtained from bioselective element (a) and the Lineweaver-Burk plot of the mentioned data (b)

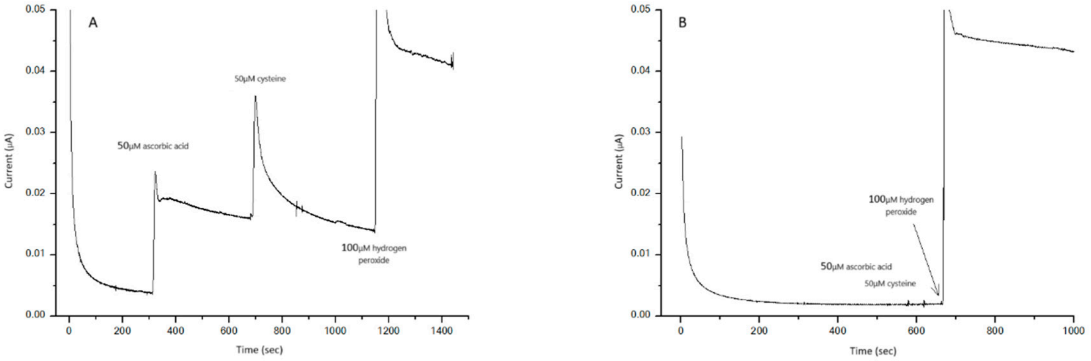

Figure S4. Responses of the biosensor without (A) and with (B) PPD membrane to the addition of interferents. Measurements were carried out in 25 mm HEPES buffer, pH 7.4, at a constant potential of +0.6 V vs Ag/AgCl reference electrode
